# Supplementary material for: Crash-perching on vertical poles with a hugging-wing robot
Source: Commun Eng. 2024 Jul 12;3:98. doi: 10.1038/s44172-024-00241-0 (PMC11245520; doi:10.1038/s44172-024-00241-0)
Supplement: Supplementary file 2 — Supplementary Information [file 44172_2024_241_MOESM2_ESM.pdf]

Supplementary Material for
Crash-perching on vertical poles with a hugging-wing robot

Mohammad Askari*, Michele Benciolini, Hoang-Vu Phan, William Stewart, Auke J. Ijspeert and Dario Floreano

*Corresponding author(s). E-mail(s): mohammad.askari@epfl.ch;

This PDF file includes:

- Supplementary Fig. S1. Inertial reorientation test setup.
- Supplementary Fig. S2. Coordinate systems and state variables.
- Supplementary Fig. S3. Static model flowchart.
- Supplementary Fig. S4. Wing segmentation and configuration selection.
- Supplementary Fig. S5. Measuring friction coefficient of vertical poles.
- Supplementary Table S1. Dynamic perching statistics with PercHug.

Other Supplementary Material for this manuscript includes:

- Video S1 (.mp4). Dynamic perching – different nose types.
- Video S2 (.mp4). Dynamic perching – wing release timing and hooks effects.
- Video S3 (.mp4). Dynamic perching – targeting and angular misalignment.

Supplementary Figures

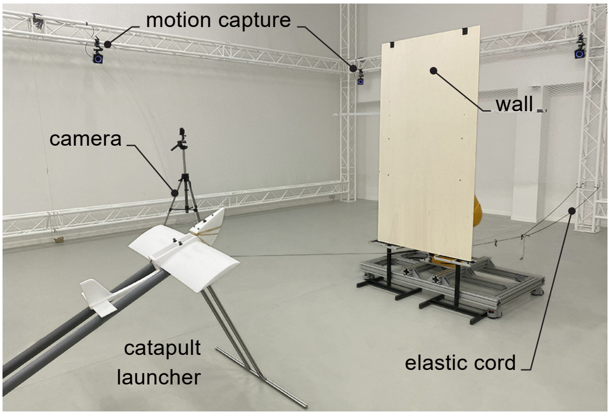

Figure S1 Inertial reorientation test setup. The setup consisted of a bungee-powered UAV catapult launcher with adjustable angle and speed for launching against a fixed vertical wall. High-speed videos were recorded using a camera, and OptiTrack motion capture system was used to capture trajectory data.

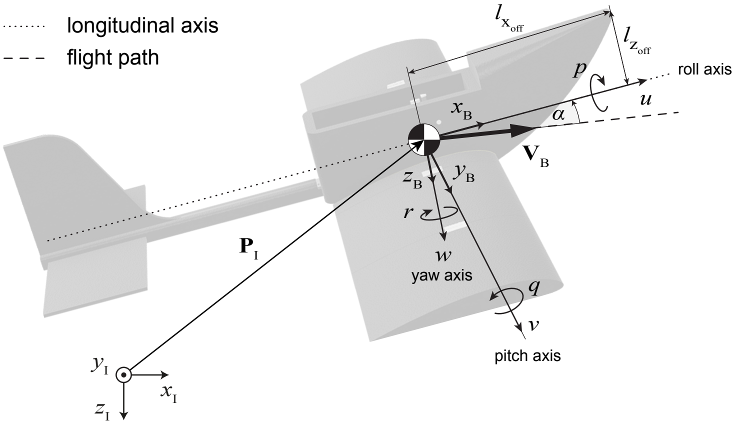

Figure S2 Coordinate systems and state variables. The black and white filled circle represents the center of gravity (COG). I and B indicate the inertial and body-attached reference frames, respectively. The 12 state variables that define the kinematics of the UAV in space are: position of the body-attached frame $\vec{\mathbf{P}}_{\mathrm{I}} = \begin{bmatrix} x & y & z \end{bmatrix}^\top$, linear velocities $\vec{\mathbf{V}}_{\mathrm{B}} = \begin{bmatrix} u & v & w \end{bmatrix}^\top$ and angular rates $\vec{\mathbf{\Omega}}_{\mathrm{B}} = \begin{bmatrix} p & q & r \end{bmatrix}^\top$ along the body-attached axes, and the body orientation based on attitude angles (roll ϕ, pitch θ, and yaw ψ, which are not shown for the sake of clarity). The angle of attack is indicated by α. The offset values $l_{\mathrm{x_{off}}}$ and $l_{\mathrm{z_{off}}}$ represent the location of the nose tip relative to the COG.

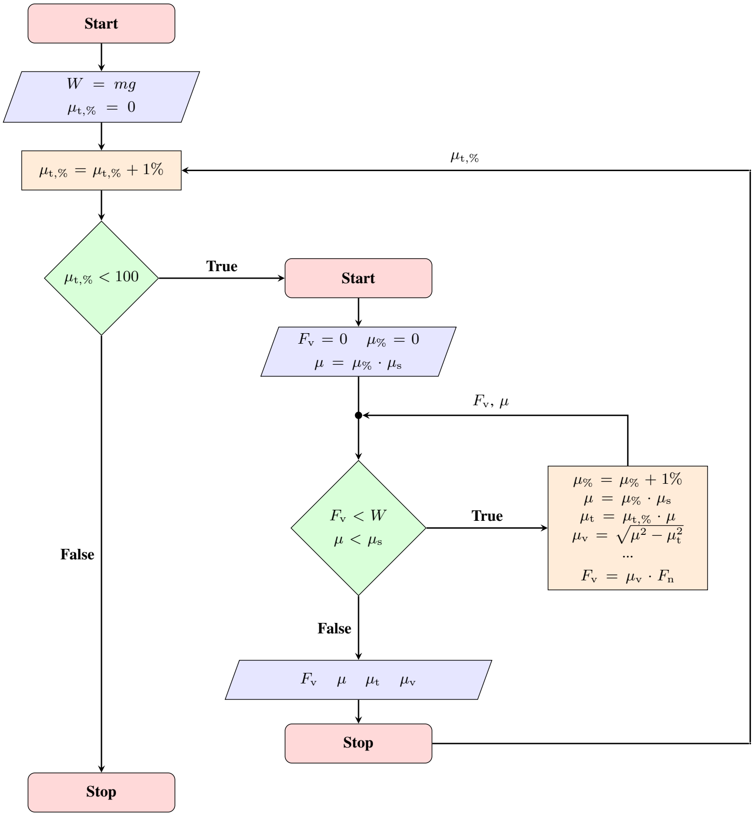

Figure S3 Static model flowchart. The iterative process that the model follows to find the division of friction between the horizontal and vertical directions.

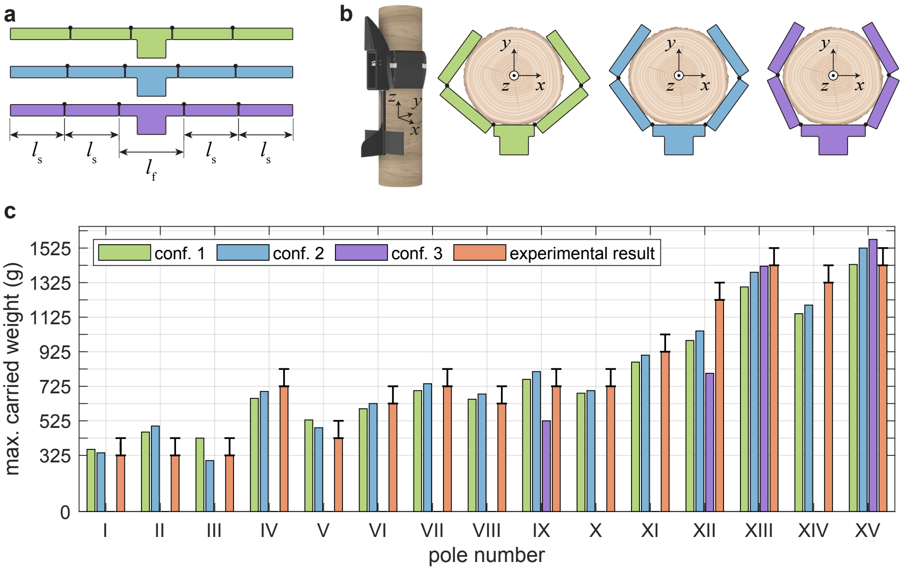

Figure S4 Wing segmentation and configuration selection. a Three different wing segmentation for a fixed wingspan of 960 mm, with two equally-sized folding segments per wing. These configurations are, namely, a narrow to a mid-range to a wide fuselage with respective widths of 140 mm, 180 mm, and 220 mm for the fuselage and fixed segments (l_{f}), and corresponding folding segment widths (l_{s}) of 205 mm, 195 mm, and 185 mm. **b** Top view illustration of the three wing configurations perched on a 340 mm pole. **c** Simulation results of static payload capacity for the three different wing configurations on the poles used in the static perching experiments (see Fig. 5 in the main text). The experimental data correspond to the selected wing design for PercHug, i.e., configuration 2.

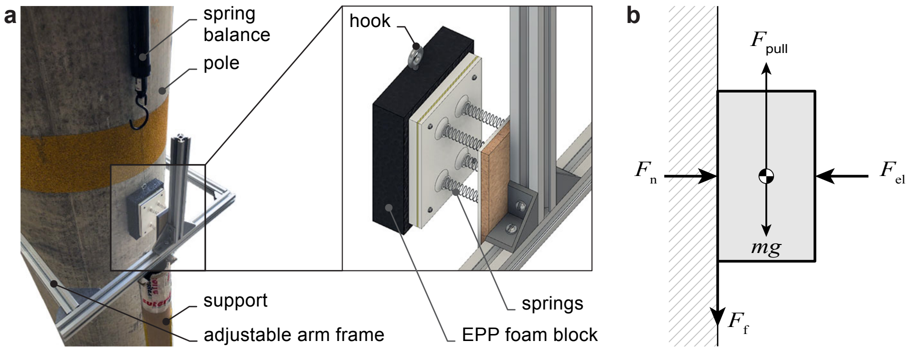

Figure S5 Measuring friction coefficient of vertical poles. a A photo and a close-up computer-aided design (CAD) view of the friction coefficient measuring tool used on a concrete pole. **b** Side view free body diagram of the EPP foam block representing the method employed for estimating the static friction coefficient.

Supplementary Table

Table S1 Dynamic perching statistics with PercHug. The table shows perching outcomes on different trees, characterized by their diameters ∅ and static friction coefficients μ_s. The presented data includes perching success rates, expressed as percentages and actual number of experiments conducted, with the standard upturned and the extended elastic nose configurations. Additionally, the average timing metrics for triggering and wrapping events are provided, measured from the impact instance.

tree	∅ (mm)	μ_s	success rate standard		success rate elastic		triggering (ms) standard	triggering (ms) elastic	wrapping (ms) standard	wrapping (ms) elastic
X	360	0.77	50 %	(3/6)	38 %	(5/13)	26 ± 5	39 ± 19	175 ± 33	163 ± 28
XI	320	0.83	75 %	(3/4)	25 %	(1/4)	30 ± 6	42 ± 14	146 ± 18	150 ± 0
XII	280	0.85	100 %	(4/4)	86 %	(6/7)	24 ± 5	31 ± 12	119 ± 16	138 ± 13
XIII	265	0.98	100 %	(4/4)	25 %	(1/4)	23 ± 7	42 ± 10	164 ± 21	200 ± 0
XIV	350	1.02	67 %	(2/3)	67 %	(2/3)	26 ± 6	24 ± 6	150 ± 6	131 ± 9
XV	270	1.04	60 %	(3/5)	14 %	(1/7)	27 ± 10	40 ± 15	192 ± 19	171 ± 0
			73 %	(19/26)	42 %	(16/38)	26 ± 7	37 ± 15	156 ± 30	152 ± 25
