# Supplementary material for: Crash-perching on vertical poles with a hugging-wing robot
Source: Commun Eng. 2024 Jul 12;3:98. doi: 10.1038/s44172-024-00241-0 (PMC11245520; doi:10.1038/s44172-024-00241-0)
Supplement: Supplementary file 3 — Description of Additional Supplementary Files [file 44172_2024_241_MOESM3_ESM.pdf]

Description of Additional Supplementary Files

File name: Supplementary Video S1

Description: Dynamic perching – different nose types. This video showcases successful perching experiments with PercHug on various trees, using the standard upturned and extended elastic nose types.

File name: Supplementary Video S2

Description: Dynamic perching – wing release timing and hooks effects. This video shows the effects of unlatching strategy (Primary vs. secondary impact release) and hooks effectiveness on perching success.

File name: Supplementary Video S3

Description: Dynamic perching – targeting and angular misalignments. This video highlights how mistargeting, lateral errors, and angular misalignments in the approach trajectory can result in perching failure.
